# Assessing the risk: Scoring systems for outcome prediction in emergency laparotomies

**DOI:** 10.7603/s40681-015-0020-y

**Published:** 2015-11-28

**Authors:** Deb Sanjay Nag

**Affiliations:** Department of Anaesthesiology & Critical Care, Tata Main Hospital, 831001 Jamshedpur, India

**Keywords:** Emergency, Laparotomy, Risk assessment, Mortality, Scoring methods

## Abstract

Emergency laparotomy is the commonest emergency surgical procedure in most hospitals and includes over 400 diverse surgical procedures. Despite the evolution of medicine and surgical practices, the mortality in patients needing emergency laparotomy remains abnormally high. Although surgical risk assessment first started with the ASA Physical Status score in 1941, efforts to find an ideal scoring system that accurately estimates the risk of mortality, continues till today. While many scoring systems have been developed, no single scoring system has been validated across multiple centers and geographical locations. While some scoring systems can predict the risk merely based upon preoperative findings and parameters, some rely on intra-operative assessment and histopathology reports to accurately stratify the risk of mortality. Although most scoring systems can potentially be used to compare risk-adjusted mortality across hospitals and amongst surgeons, only those which are based on preoperative findings can be used for risk prognostication and identify high-risk patients before surgery for an aggressive treatment. The recognition of the fact, that in the absence of outcome data in these patients, it would be impossible to evaluate the impact of quality improvement initiatives on risk-adjusted mortality, hospital groups and surgical societies have got together and started to pool data and analyze it. Appropriate scoring systems for emergency laparotomies would help in risk prognostication, risk-adjusted audit and assess the impact of quality improvement initiative in patient care across hospitals. Large multi-centric studies across varied geographic locations and surgical practices need to assess and validate the ideal and most apt scoring system for emergency laparotomies. While APACHE-II and P-POSSUM continue to be the most commonly used scoring system in emergency laparotomies,studies need to compare them in their ability to predict mortality and explore if either has a higher sensitivity and specificity than the other.

## 1. Introduction

Emergency laparotomy is most common emergency surgery and describes an exploratory procedure for which the clinical presentation, underlying pathology, anatomical site of surgery, and perioperative management vary considerably. The fact that over 400 different surgical procedures have been recorded during an emergency laparotomy, reflect the diverse nature of this surgical cohort [1]. The varied surgical pathology and the emergent nature of the procedure limits the time to optimize these patients [1]. Although there is scarcity of outcome data after emergency laparotomy, it is generally recognized to be poor [1]. Even after innumerable advances in surgical skills, antimicrobial agents and supportive care, the mortality of peritonitis remains high, and is presently reported to be between 14.9-19.5% [1, 2].

Early prognostic evaluation of these patients is desirable to select the high-risk patients for a more aggressive treatment [3]. The continuous monitoring and audit of clinical practice is an essential part of making improvements in medical science and enhancing patient care [4]. It is also essential to ensure that patients are well informed of risks and to improve quality of care in hospitals.

Knowing which patient is at risk of developing complications or dying contributes to the quality of surgical care and cost reduction [5]. Doctors are legally bound to inform their patients of the potential risks involved with a particular treatment [5-7]. It is therefore essential to identify and make appropriate decision on those patients who are at high-risk of developing serious complications or die [5, 7-9]. Categorizing patients into different risk groups would also help prognosticate the outcome, select patients for intensive care and determine operative risk, thereby helping to choose the nature of the operative procedure, e.g. damage control vs. definitive procedure.

An ideal scoring system is desirable, so that an accurate prediction of outcome could then be made, allowing the treating team to present a more informed choice to the patient on whether surgery or supportive care is the optimal management [10]. It should also allow analysis for risk-adjusted comparison between surgeons, hospitals and across geographical distributions [11].

## 2. The scoring systems

Various scoring systems are available to predict surgical outcomes. They range from the general scoring systems to surgery specific scoring systems. While most scoring systems compare post-operative mortality as an outcome parameter, some are also designed to predict morbidity. While American College of Surgeons recommend the Universal ACS NSQIP Surgical Risk Calculator [12] for mortality and morbidity risk assessment for informed consent and to facilitate decision making for patients and surgeons, P-POSSUM scoring system was used to assess improved outcomes in patients undergoing emergency laparotomy after the implementation of emergency laparotomy pathwayquality improvement care (ELPQuiC) bundle [13]. While those which can calculate the risk based on pre-operative parameters are most useful in prognostication and triage of patients, those that need intra-operative data are best utilized for retrospective quality audits. While many scoring systems have been used in emergency laparotomies, till date no specific scoring system has been developed for emergency laparotomies.

## 3. The general scoring systems

### 3.1. ASA

The oldest available scoring system [14], the American Society of Anesthesiologists’ (ASA) physical status score is often used to subjectively estimate preoperative health status. While it was initially designed for “statistical data collection and reporting”, it is today used to predict the perioperative risk [15]. This subjective scoring is associated with inter-observer variability [14] and has “no specific role” [15] in predicting the outcome in emergency laparotomy. The ASA Scores range from ASA I for a normal healthy patient, ASA II for a patient with mild systemic disease, ASA III for a patient with severe systemic disease, ASA IV for a patient with a severe systemic diseas that is a constant threat to life and ASA V for a moribund patient who is not expected to survive without the operation. An addition of E besides the ASA Grade denotes emergency surgery. A study on 10864 patients using receiver-operating characteristic (ROC) curve area, Sankar A *et al*. found that its ability to predict mortality (ROC Curve area 0.69) and cardiac complications (ROC Curve area 0.70) was only moderate [14]. However, the ASA Score is simple, able to predict mortality as well as morbidity in general surgical patients, and has also been able to predict mortality well in a particular subset of patients undergoing emergency laparotomy, those with peptic ulcer perforation [15].

### 3.2. Apgar score for surgery

It is a 10 point score considering the estimated amount of blood loss, lowest heart rate, and lowest mean arterial pressure. Scores of less than or equal to 4 was associated with significant higher mortality [16]. However it can only be calculated at the end of surgery. This scoring system can has been reported to predict death with a significant degree of accuracy (*P* = 0.0001 in univariate univariate logistic regression, c-statistic 0.92) [16]. Scores of 9-10 was associated with 0% death, 7-8 with 0.3% mortality, 5-6 with 4.9% mortality and 0-4 was associated with a mortality of 13.8% [16]. This scoring system was also able to predict major complications with a only 4% complications scores with scores of 9-10 and a 50% risk of major complications with a score of 4 [16]. It has beentested mostly in general surgical and vascular surgeries, and not been validated to specifically assess the risk in emergency laparotomies [16].

### 3.3. Sickness assessment (SA)

It was essentially used to predict mortality in the geriatric population undergoing emergency surgery. It is a simple scoring system using only three parameters at the time of admission, hypotension, pre-existing severe chronic disease and whether or not the patient was functionally independent [17]. In patients above 65 years undergoing emergency laparotomy, an SA Score of 1 was associated with a 52% mortality, a score of 2 was associated with 60% mortality and a score of 3 was associated with 100% mortality [17]. The mortality was 15% in those with SA score as zero [17]. Although it is a simple scoring system to identify the high risk group, its utility in large multi-centric studies or across agegroups has not been evaluated till date. Kennedy RH et al found that SA Scores had significant predictive ability (*P* < 0.001) in predicting mortality and the APACHE II scoring system was not superiorto it [17]. No study till date has evaluated its correlation with peri-operative morbidity.

### 3.4. Calculation of post-operative risk in emergency surgery (CORES)

The Calculation of post-Operative Risk in Emergency Surgery (CORES) [18] was constructed based on a regression model and needs only 6 preoperative variables to predict the in-hospital mortality. The predicted mortality is calculated using an equation based on presence or absence of (1) Japan Coma Scale >30, (2) ASA Class3, (3) ASA Class 4, (4) Whie blood cell count of <2,500 cells/μL, (5) platelet count<150,000 or>300,000 cells/μL and (6) blood urea nitrogen >/=40 mg/dL [18]. After development, its accuracy was further assess on 1471 cases across six hospitals and found to be as discriminative as P-POSSUM [18]. In predicting in-hospital mortality, the area under the receiver operating characteristic curve (AUC) (95% CI) of the CORES model was high (0.85), the observed-to-estimated mortality ratio (OE ratio) was also high (0.70), and the calibration power was also good (chi-square = 19.9, degrees of freedom = 8, *P* = 0.81) [18]. Although this model was developed to specifically predict mortality, the CORES scores were also significantly correlated to other post-operative morbidity [18]. While this was the first, and possibly only specific model predicting the post-operative risk for emergency surgery, it is yet to be validated specifically on emergency laparotomies [18].

### 3.5. Estimation of physiologic ability and surgical stress (E-PASS)

The Estimation of Physiologic Ability and Surgical Stress (EPASS) scoring system aims to quantify the patient’s reserve and surgical stress was initially developed to predict morbidity and mortality in elective gastrointestinal surgery [19]. They have been subsequently been evaluated in emergency gastrointestinal study and was “useful for assessing the risks of emergency abdominal surgery” [20].

This system uses a preoperative risk score (PRS) and a surgical stress score (SSS) to calculate a comprehensive risk score (CRS) [19]. PRS is calculated from a formula using age, presence or absence of severe heart-disease, severe pulmonary disease and diabetes mellitus, along with performance status index (based on the definition by Japanese Society for Cancer Therapy) and American Society of Anesthesiologists physiological status classification [19]. Surgical stress score (SSS) is calculated by an equation based on blood loss/body weight (mL/kg), operation time (in hours), and extent of skin incision [19].

With increase in CRS, there was a significant increase in incidence of postoperative morbidity and mortality (*P* < 0.0001) [19]. With CRS <0.1 the morbidity and mortality was 12.5% and 0%, respectively [19]. With CRS 0.5-0.75, the morbidity and mortality rates were 45.0% and 5.0%, respectively, and when the CRS > 1.0, the morbidity and mortality rates were 76.9% and 38.5%, respectively [19]. However it is yet to be validated in large multicentric trials.

### 3.6. ACS NSQIP surgical risk calculator

American College of Surgeons National Surgical Quality Improvement Program (ACS NSQIP) Surgical Risk Calculator [13] is based on 21 preoperative risk factors identified from 1,414,006 patients across 393 ACS NSQIP hospitals in the United States. It also allows a Surgeon Adjustment Score to reasonably the modify the score based on their clinical impression. This scoring system can be used for over 1500 procedures across surgical specialties. It can specifically predict the risk for 10 different Current Procedural Terminology (CPT) codes for laparotomies. CPT code is the descriptive terms for identifying and reporting medical services and procedures in the United States. Apart from mortality, it is able to predict the risk of serious or any other post-operative complication including pneumonia, cardiac complication, surgical site infection, urinary tract infection, venous thromboembolism, renal failure or return to the operating room. Although the discriminative ability has been found to be reasonable accurate as compared to other risk calculators [13], it is yet to be accurately evaluated specifically for emergency laparotomies. Besides, it is yet to be evaluated across geographical locations and the Surgeon Adjustment Score is based on subjective assessment with “no quantitative evidence that these adjusted risks are more accurate” [13].

Other surgical risk assessment tools like the Charlson Comorbidity Index validated in over 5500 studies since 1987 for its ability to predict mortality based on co-morbidity [21], the Fitness Score [10] in major abdominal surgery (including emergency surgery) and Reiss Index [10] used specifically in laparotomies in elderly patients have all been used to assess the surgical risk. However, none of the scoring system has been specifically been assessed in patients undergoing emergency laparotomies across all age groups.

## 4. The critical care and sepsis scoring systems

### 4.1. APACHE II

The original APACHE score based on physiology score for acute illness and chronic health status was developed in 1981 by Knaus WA *et al*. [22] and was subsequently simplified to create APACHE II in 1985 [23]. The score is calculated based on the patient’s age, 12 routine physiological measurements and whether they are scheduled for routine or emergency surgery. An integer score from 0 to 71 is computed based on these values; higher scores are associated with an higher risk of death [23]. Till date, this remains the most widely used illness severity score worldwide [24]. It has been evaluated to predict mortality in patients undergoing in general surgical or patients undergoing laparotomy since 1990. Till date, at least 14 studies [25-38] (Table 1) have tried to correlate the APACHE II with the risk of mortality and re-exploration.

In the study by Oka Y *et al*. in patients with peritonitis undergoing laparotomy, APACHE II scores amongst survivors ranged from 0-21 (Mean 5.0) , with a mean of 5.0 [30]. Amongst those patients who died, the scores ranged from 15-38 (Mean 23.3) [30]. The difference between groups was significant (*P* < 0.05) [30]. The co-relation with morbidity was not reported in this study.

Another study by Scheien M *et al*. in patients with perforated peptic ulcer reported zero mortality in patients with scores below 11 points and 35% mortality rate in patients with APACHE II scores above 11 [25]. In other studies by Lee FY *et al*. also, APACHE II was found to predict both morbidity and mortality but AUC or relative risks were not reported in this study [36].

### 4.2. Simplified acute physiology score (SAPS), multiple organ dysfunction score (MODS), sepsis-related organ failure assessment (SOFA) score, sepsis score, multiple organ failure (MOF) score

While SAPS, MODS and SOFA Scores were able to predict mortality in peritonitis patients, they were unable to predict “ongoing infection needing a relaparotomy” [34]. The SAPS II score is calculated from 12 physiological and 3 disease-related variables. The SAPS II score ranges from 0 to 163 points. A MODS is calculated from 0-4 scoring for each of respiratory, hematologic, hepatic, cardiovascular, Glasgow Coma Scale and renal parameters. The scores range from 0-24.

The SOFA Score is also calculated from 0-4 scoring for each of respiratory, cardiovascular, nervous, hepatic, coagulation and renal systems with scores ranging from 0-24. The MOF Score is derived from 0-2 score (0: Normal function, 1: Organ dysfunction, 2: Organ failure) for each of respiratory, cardiovascular, renal, hepatic, hematologic, gastrointestinal and the central nervous systems with scores ranging from 0-14. Higher scores are associated with increased risk of morbidity and mortality.

The ability of the scoring system to predict mortality was assessed using the area under the receiver operating characteristic (ROC) curve (AUC) by Oddeke van Ruler et al. and they found it to be 0.8 for SAPS-II, 0.72 for SOFA (day 1) and 0.76 for MODS (day 1) [34]. In their study, the authors found the ability of this scoring system to be statistically significant in all the 3 scoring systems [34]. Other morbidity was not studied in this trial but this study failed to demonstrate the correlation of these scoring systems to predict relaparotomy [34]. Similarly, the Sepsis Score and Multiple Organ Failure (MOF) Score may also predict mortality, but has not been extensively studied in patients undergoing emergency laparotomy.

### 4.3. Mannheim peritonitis index (MPI)

The MPI based on retrospective analysis of 1253 patients with peritonitis has 8 proven risk factors based on their predictive power [39]. MPI scores of pre were associated with a mortality of 5%, scores of 21-29 had a mortality of 14% and scores ≥ had a mortality of 14% 50% [39]. MPI score of 25 had sensitivity and specificity of 72.09% and 71.43% respectively in predicting mortality, and 80.65% and 57.89% sensitivity and specificity respectivelyfor predicting morbidity [39]. Other studies also observed strong association between increasing MPI score and adverse outcomes in patients with secondary peritonitis [40].

**Table 1:** APACHE II scoring for outcome in emergency general surgery or laparotomy.

Year	Patient Category	Outcome
1990	Perforated peptic ulcers	APACHE II scoring system accurately stratified patients according to risk [25]
1997	Peritonitis and intra abdominal sepsis	Combination of the APACHE II and the MPI provides the best scoring system [26]
2007	Peritonitis due to hollow viscus perforation	APACHE-II scoring system can be used to assess group outcomes in patients with peritonitis due to hollow viscus perforation [27]
2007	General surgical patients	M-POSSUM is more accurate than POSSUM and APACHE II in predicting postoperative morbidity and mortality [28]
2007	Perforated peptic ulcer	Compared to the APACHE II & III & the simplified acute physiology score II, the mortality probability models (MPM) II predicted mortality at admission better [29]
2010	Patients with peritonitis	APACHE II is accurate in predicting mortality has definitive advantages and is therefore more useful [30]
2010	Generalized secondary peritonitis	Independent mortality predictors were APACHE II > or = 16 [31]
2011	obstructing colon cancer	APACHE II score ≥11 was a prognostic factor for poor outcome [32]
2011	Perforation peritonitis	APACHE II is superior in prediction of the outcome as compared to SAPS I, Sepsis score, MOF, TISS-28 and MPI [33]
2011	Abdominal sepsis that have ongoing infection and would need relaparotomy	All evaluated scoring systems (APACHE-II score, SAPS-II, Mannheim Peritonitis Index (MPI), MODS, SOFA score, and the acute part of the APACHE-II score) were predictive of mortality, none predicted need for laparotomy [34]
2012	Secondary peritonitis of colorectal origin to predict relaparotomies	APACHE II score might be helpful in predicting the need for relaparotomies [35]
2013	Perforated peptic ulcer	APACHE II has been shown to predict outcome well also for PPU patients [36]
2014	Patients of intra-abdominal sepsis and treated with planned relaparotomy	APACHE II scoring system is reliable for prediction of mortality [37]
2015	Gall bladder perforation	Both POSSUM and APACHE II scores were superior to ASA score in risk prediction [38]

## 5. The disease specific scoring systems

### 5.1. Perforated peptic ulcer scoring systems: boey score, Hacettepe score, Jabalpur score and the peptic ulcer perforation (PULP) score

The Boey score was the first developed scoring system to predict mortality in perforated peptic ulcer in 1982 [41]. Subsequent studies in 1987 validated the value of the three independent variables in this scoring system, severe medical illness, pre-existing shock and longstanding perforation in predicting mortality [42]. Risk score 0 was not associated with any mortality, risk score 1 was associated with a mortality of 10%, Risk score 2 was associated with a mortality of 45.5% and a Risk score 3 was associated with a mortality of 100%. The accuracy rate in predicting mortality was 93.9% and there were no false negative errors [42].

The Hacettepe score uses coexisting medical illness, acute renal failure, raised white cell count and male sex as the 4 variables for predicting mortality with mortality increasing with rising scores. When developed in 1992, “the sensitivity was 83%, the specificity 94%, and the overall predictive accuracy 93%.” [43]. However it has not been found to be better than other scoring systems used to predict outcome in peptic ulcer perforation [36].

The Jabalpur score used multiple regression analysis and developed a scoring system based on six identified risk factors which included age, co-morbid illness, perforation to surgery time interval, preoperative shock, heart rate and serum creatinine levels to predict post-operative mortality [44]. Scores of 0-4 was associated with 14% morbidity and 0% mortality, scores of 5-9 was associated with 48% morbidity and 7% mortality, scores of 10-14 were associated with 71% morbidity and 38% mortality, while scores of 15-21 was associated with 100% morbidity and mortality [44]. There was good correlation of the Jabalpur score in predicting both morbidity and mortality with correlation coefficient being 0.67 (*P* < 0.001) and 0.81 (*P* < 0.001) for mortality and morbidity respectively [44].

The Peptic Ulcer Perforation (PULP) score is based on age, presence of comorbid diseases, concurrent use of steroids, shock on admission, serum creatinine levels, time from perforation to admission and ASA scores [45]. Scores of 0-7 was associated with low risk (<25%) and scores of 8-18 was associated with high risk (>25%) of mortality [45]. Its accuracy to predict mortality by the area under receiver operating characteristics curve (AUC) was better (AUC 0.83 for PULP) than the Boey score (AUC 0.70) and ASA score (AUC 0.78) [45]. This study did not analyze other post operative morbidity.

### 5.2. Patients with Liver Disease: Child-Turcotte-Pugh (CTP) classification&MELD (model for end stage liver disease) score

Nearly 10% of the patients undergoing surgery have some form of liver disease. The CTP classification is based on serum albumin and bilirubin levels, prothrombin time, and the degree of encephalopathy and ascites. In major abdominal surgery, the mortality in Child’s class A was reported to be 10%, 30-31% in class B and 76-82% in class C [46]. It also correlated well with postoperative complications including “liver failure, worsening encephalopathy, bleeding, infection, renal failure, hypoxia and intractable ascites” [46].

Although the MELD was initially designed to predict mortality after Transjugular intrahepatic portosystemic shunt (TIPS), it is based on a linear regression model, assessing the risk from a score derived from the patient’s creatinine level, serum bilirubin and International Normalized Ratio of the Prothrombin Time (PT/INR) [46]. It has been shown to prognosticate the risk of mortality in patients undergoing abdominal, orthopedic and cardiac surgery with 30-day mortality ranging from 5.7% for a MELD score </= 7, 10.3% for MELD Score of 8-11 and 25.4% for MELD score of 12-15 with emergency surgery being an independent predictor of the duration of hospital stay [47]. The risk of mortality increased linearly for MELD Scores above 8.

However, none of the scoring systems have been studied to stratify the risk of morbidity or mortality in patients undergoing emergency laparotomy. Possibly, this subset of patients with liver disease, depending on the preexisting hepatic insult, the hemodynamic instability and the nature of surgery, would be at a much higher risk as compared to those without pre-existing liver disease. The existing scoring system needs to be further validated in these patients.

### 5.3. Colorectal Surgery: AFC-index and Cleveland clinic colorectal cancer model

Although the APACHE, ASA or POSSUM or its modified form for colorectal surgery have been most commonly been used in these subset of patients in whom the survival has increased substantially over the last 25 years, the French Association for Surgery (Association Française de Chirurgie, AFC) identified independent factors leading to death by multivariate logistic regression analysis and developed the AFC-index [48]. The four independent preoperative risk factors,namely emergency surgery, loss of more than 10% of weight, neurological disease history and age > 70 years have been shown to predict mortality with the same sensitivity and specificity as P-POSSUM [48]. An AFC Score of 0 was associated with a mortalityof 0.5%, score of 1 with a mortality of 1.6%, score of 2 with a mortality of 7.2%, score of 3 with a mortality of 46.8% and a score of 4 was associated with a mortality of 70% [48]. Although the study by Arnaud Alves *et al*. did report a postoperative morbidity of 23% in 239 studied patients, the did not establish any correlationwith the AFC-index [48].

The Cleveland clinic colorectal cancer model was developed using a multilevel Bayesian logistic regression model and it identified age, ASA grade, TNM Staging, urgent need for surgery, cancer resection status and the hematocrit levels as independent risk factors for mortality [49]. The model offered excellent correlation between the observed and predicted mortality and an area under receiver operator characteristic (ROC) curve (AUC) of 0.801 [49].

## 6. Surgical audit scoring systems

### 6.1. Possum & its varients

Copeland *et al*. first described POSSUM (Physiological and Operative severity for the enumeration of mortality and morbidity) in 1991 as a scoring system for surgical audit [8]. They used logistic regression analysis to predict both morbidity and mortality. However, it was found to over predict death, especially amongst the low risk patients [50]. This led to the modification of the logistic regression and development of the Portsmouth POSSUM (P-POSSUM) [50]. P-POSSUM used the same physiological and operative scoring methods initially described by Copeland *et al*. and its predicted mortality matched with the observed mortality [51]. It uses 12 physiological and 6 operative parameters which were divided into 4 grades with exponentially increasing score (1, 2, 4, and 8) to calculate the risk of mortality. The minimum score is 12 and maximum score is 88, with higher scores predicting higher mortality.

POSSUM has subsequently been modified for application in various types of surgeries, O-POSSUM for orthopedic surgeries [52, 53], V-POSSUM for vascular surgeries [54] and Cr-POSSUM for colorectal surgeries [55].

P-POSSUM still remains the scoring system of choice for general surgeries and also for emergency laparotomies, especially in the United Kingdom. Numerous studies have validated POSSUM or one of its variants in general surgery, laparotomy or in high risk patients (Table 2 [56-67]).

P-POSSUM has emerged as the most dependable scoring system for audit purposes and for evaluating the impact of quality improvement initiatives across the United Kingdom in patients undergoing emergency laparotomy. In a recent multicentre study across four National Health Service (NHS) hospitals, ELPQuiC bundles (Table 3) brought about a significant reduction in PPOSSUM risk-adjusted 30-day mortality in patients undergoing [13].

Sreeharsha H *et al*. used linear analysis for comparing the observed and predicted mortality using POSSUM. The observed to predicted ratio (O: P) was 0.71 and there was “no statistically significant difference between the predicted and observed values” [67]. An O:P ratio of 1.19 suggested that there was no significant difference between the observed and predicted morbidity also. Chieng *et al*. observed P-POSSUM (O: P Ratio 0.721) to be a “better scoring system” compared to POSSUM (O: P Ratio 0.366) [11].

### 6.2. Surgical mortality probability model (S-MPM)

The S-MPM [68] is a 9-point 30-day mortality risk index. Patients were assigned points as per ASA Status (O for ASA I, 2 for ASA II, 4 for ASA III, 5 for ASA IV and 6 for ASA V), risk of surgery (1 for intermediate risk and 2 for high-risk procedures) and for emergency surgery (1 point). A total risk score <5 was associated with 0.5% mortality, score of 5-6 with 1.5-4% mortality and scores >6 with 10% mortality.

While this simple scoring system can be easily calculated at the bed-side and “used by surgeons andhospitals to internally audit their quality of care”, it can also help risk-prognostication and prioritization of patients [68]. This scoring system is fairly accurate as compared to ACS NSQIP mortality model with “slightly worse discrimination and marginally better calibration” [68]. S-MPM using only 3 predictors had an ability for discrimination with CStatistic (which predicts that outcome is better than chance) of 0.90 as compared to the ACS NSQIP risk adjustment model using 35 variables which has a C-Statistic of 0.94. This model has only been applied on limited types of procedures and its validity specifically in emergency laparotomy remains to be ascertained.

**Table 2:** Use of POSSUM or one of its variants in general surgery, laparotomy or high risk surgical patients.

Year	Patient Category	Outcome
2004	Patients needing damage control laparotomy	Lower mortality than that predicted by P-POSSUM and POSSUM with Damage Control Surgery [56]
2004	Patients undergoing emergency laparotomy	POSSUM is a good predictor of morbidity and mortality. P-POSSUM predicts mortality equally well. Both can be used for risk-adjusted surgical audit [57]
2005	High risk patients undergoing surgery	p-POSSUM predicted mortality well but POSSUM over-predicted mortality [58]
2006	elective and emergency laparotomy	It is a useful predictor of morbidity and mortality [59]
2007	General surgery	M-POSSUM correlates better with postoperative complications and mortality than POSSUM [60]
2008	cases of ileal perforations	Significant correlation between POSSUM score and postoperative complications and deaths [61]
2009	Patients undergoing emergency laparotomy	P-POSSUM predicts mortality better than POSSUM. Exponential method is better than linear regression analysis [62]
2009	Unresectable pancreatic cancer during exploratory laparotomy	POSSUM scoring system is an independent predictor of survival in multivariate analysis [63]
2009	oncologic gastric surgery	Mortality lower than that predicted by POSSUM and higher than that predicted by P-POSSUM [64]
2010	patients undergoing emergency surgery	ASA grade and POSSUM scores were the better predictors of mortality than EWS, APACHE II, and age [65].
2010	general surgical laparotomy	P-POSSUM is a better overall predictor of mortality than POSSUM [11].
2011	General surgical patients	Both POSSUM and P-POSSUM are valid indices for risk prediction of morbidity and mortality [66]
2012	secondary peritonitis of colorectal origin	CR-POSSUM had the highest sensitivity and specificity to predict mortality as compared to MPI & APACHE-II [35]
2014	Emergency laparotomy	POSSUM is an accurate predictor of mortality and morbidity and can be used for surgical audit [67]

**Table 3:** Evidence-based care bundle for patients undergoing emergency laparotomy [13].

Bundle	Element
1	Early warning score assessment for all emergency admissions with graded escalations
2	All patients with suspicion of peritoneal soiling or diagnosis of sepsis to receive early broad-spectrum antibiotics
3	Laparotomy within 6 hours of decision to operate
4	Goad directed resuscitation as soon as possible, or within 6 hours of admission
5	ICU admission for all patients in the immediate post-operative period

## 7. Conclusion

Although numerous scoring systems for risk prognostication (Table 4) has been developed since the ASA Score was first introduced in 1941 [68], none have been able to comprehensively achieve the goals of being easy to calculate, fairly accurate in its prediction, reproducible across geographical locations, able to audit surgical outcomes across hospitals and assess the change brought about by any quality improvement initiatives.

Emergency laparotomies remain the commonest emergency surgery across most hospitals continue to be associated with significantly higher mortality as compared to most other major general surgical procedures or elective surgeries [1]. Therefore, a scoring system is not only necessary to predict mortality in this category of patients, initiative resulting in improved quality of care should also reflect on the surgical risk adjusted mortality. Emergency laparotomies involves “considerable cost” [69] to the healthcare providing agencies or the individual, either directly or through insurance. Similarly, quality improvement initiatives like availability of operation theatre space or consultant coverage round the clock would also involve financial implications. Therefore studies like those Huddart S. *et al*. (on behalf of the ELPQuiC Collaborator Group) [38] which showed that evidence based care bundles (Table 3) saved 8.11 lives per 100 patients treated, could also justify the efforts to enhance the quality of care and also its financial impact. Besides, it would also help the medical fraternity in identifying those quality improvement initiatives which actually bring about a risk-adjusted benefit, as compared to those, which in absence of consensus, are being practiced merely based on individual perception [70].

**Table 4:** Scoring systems for emergency laparotomy.

Scoring System		Preoperative risk evaluation possible
General Scoring Systems		
	ASA	Yes
	Apgar Score for Surgery	No
	Sickness Assessment (SA)	Yes
	Calculation of post-Operative Risk in Emergency Surgery (CORES)	Yes
	Estimation of Physiologic Ability and Surgical Stress (E-PASS)	No
	ACS NSQIP Surgical Risk Calculator	Yes
The Critical Care & Sepsis Scoring Systems		
	APACHE II	Yes
	Simplified Acute Physiology Score (SAPS)	Yes
	Multiple Organ Dysfunction Score (MODS)	Yes
	Sepsis-related Organ Failure Assessment (SOFA) score	Yes
	Sepsis Score	Yes
	Multiple Organ Failure (MOF) Score	Yes
	Mannheim peritonitis index (MPI)	No
The disease specific scoring systems		
Perforated Peptic Ulcer Scoring Systems	Hacettepe score	Yes
	Boey score	Yes
	Jabalpur score	Yes
	Peptic Ulcer Perforation (PULP) score	Yes
Patients with Liver Disease	Child-Turcotte-Pugh (CTP) classification	Yes
	MELD (model for end stage liver disease) score	Yes
Colorectal Surgery	AFC-index	Yes
	Cleveland clinic colorectal cancer model	No
Surgical Audit Scoring systems	POSSUM & its variants	No
	Surgical Mortality Probability Model (S-MPM)	No

Till very recently, ease of calculation, especially at the bedside, used to be an extremely essential criteria for any scoring system. However, the advent of smart phones and mobile applications have made the use of even intricate scoring systems like the APACHE-II, P-POSSUM and ACS NSQIP Surgical Risk Calculator, very simple. Today, hand-held devices like smartphones, personal digital assistants (PDAs) or tablets allows us to use complex formulas and various regression models to calculate the risk at the patient’s bedside.

APACHE-II and P-POSSUM remain the most commonly used scoring system in emergency laparotomies (Table 1 & Table 2). Although P-POSSUM has been most frequently used for audit purposes in this cohort, it is associated with certain limitations. Operative variables such as estimated blood loss or peritoneal contamination may have significant inter-observer bias. A similar surgery by two different surgeons, one causing or estimating higher blood-loss than the other, will cause a change in the observed to expected (O/E) risk ratio. Besides, the delay in getting histopathology reports can also delay the risk assessment. APACHE-II scores can however be calculated preoperatively and has been shown to correlate well with postoperative mortality. However, unlike P-POSSUM, it does not consider etiology or degree of peritoneal contaminationand is purely based on the acute physiologic and chronic health status of the patient. While it does eliminate risk assessment based on subjective evaluation of certain risks in the P-POSSUM scoring system (example, peritoneal soiling or estimated blood loss), it does not consider the surgical procedure or the operative findings. However, it does factor-in emergency surgeries while calculating the risk.

Large multicentric studies across varied geographic locations and surgical practices need to assess and validate the ideal and most apt scoring system for emergency laparotomies. Studies need to compare APACHE-II and P-POSSUM in their ability to predict mortality and explore if either has a higher sensitivity and specificity than the other. Any impact on the risk-adjusted mortality can bring about significant reduction in mortality amongst patients undergoing one of the commonest emergency surgeries worldwide.

## References

[1] Saunders DI, Murray D, Pichel AC, Varley S, Peden CJ (2012). UK Emergency Laparotomy Network. Variations in mortality after emergency laparotomy: the first report of the UK Emergency Laparotomy Network.. Br J Anaesth..

[2] Vester-Andersen M, Lundstrøm LH, Møller MH, Waldau T, Rosenberg J, Møller AM (2014). Danish Anaesthesia Database. Mortality and postoperative care pathways after emergency gastrointestinal surgery in 2904 patients: a population-based cohort study. Br J Anaesth.

[3] Ahuja Ashish, Pal Ravinder (2013). Prognostic scoring indicator in evaluation of clinical outcome in intestinal perforations. J Clin Diagn Res.

[4] Mercer SJ, Guha A, Ramesh VJ (2013). The P-POSSUM scoring systems for predicting the mortality of neurosurgical patients undergoing craniotomy: further validation of usefulness and application across healthcare systems. Indian J Anaesth.

[5] Neary WD, Heather BP, Earnshaw JJ (2003). The Physiological and Operative Severity Score for the enumeration of Mortality and morbidity. Br J Surg.

[6] Knaus W, Wagner D, Draper E (1991). The APACHE III prognostic system. Risk prediction of hospital mortality for critically ill hospitalized adults. Chest.

[7] Chang R (1989). Individual outcome prediction models for predictive care unit. Lancet.

[8] Copeland GP, Jones D, Walters M (1991). POSSUM: A scoring system for surgical audit. Br J Surg.

[9] Bann SD (2001). Comparative Audit: The trouble with POSSUM. JR Soc Med.

[10] Rix TE, Bates T (2007). Pre-operative risk scores for the prediction of outcome in elderly people who require emergency surgery. World J Emerg Surg.

[11] Chieng TH, Roslan AC, Chuah JA (2010). Risk-adjusted analysis of patients undergoing laparotomy using POSSUM and P-POSSUM score in Queen Elizabeth Hospital, Sabah. Med J Malaysia.

[12] Bilimoria KY, Liu Y, Paruch JL, Zhou L, Kmiecik TE, Ko CY (2013). Development and evaluation of the universal ACS NSQIP surgical risk calculator: a decision aid and informed consent tool for patients and surgeons. J Am Coll Surg.

[13] Huddart S, Peden CJ, Swart M, McCormick B, Dickinson M, Mohammed MA (2015). ELPQuiC Collaborator Group; ELPQuiC Collaborator Group. Use of a pathway quality improvement care bundle to reduce mortality after emergency laparotomy. Br J Surg.

[14] Sankar A, Johnson SR, Beattie WS, Tait G, Wijeysundera DN (2014). Reliability of the American Society of Anesthesiologists physical status scale in clinical practice. Br J Anaesth.

[15] Thorsen K, Søreide JA, Søreide K (2013). Scoring systems for outcome prediction in patients with perforated peptic ulcer. Scand J Trauma ResuscEmerg Med.

[16] Gawande AA, Kwaan MR, Regenbogen SE, Lipsitz SA, Zinner MJ (2007). An Apgar score for surgery. J Am Coll Surg.

[17] Kennedy RH, al-Mufti RA, Brewster SF, Sherry EN, Magee TR, Irvin TT (1994). The acute surgical admission: is mortality predictable in the elderly?. Ann R CollSurg Engl.

[18] Miyazaki N, Haga Y, Matsukawa H, Ishimura T, Fujita M, Ejima T (2014). The development and validation of the Calculation of post-Operative Risk in Emergency Surgery (CORES) model. Surg Today.

[19] Oka Y, Nishijima J, Oku K, Azuma T, Inada K, Miyazaki S (2005). Usefulness of an estimation of physiologic ability and surgical stress (E-PASS) scoring system to predict the incidence of postoperative complications in gastrointestinal surgery. World J Surg.

[20] Koushi K, Korenaga D, Kawanaka H, Okuyama T, Ikeda Y, Takenaka K (2011). Using the E-PASS scoring system to estimate the risk of emergency abdominal surgery in patients with acute gastrointestinal disease. Surg Today.

[21] Quan H, Li B, Couris CM, Fushimi K, Graham P, Hider P (2011). Updating and validating the Charlson comorbidity index and score for risk adjustment in hospital discharge abstracts using data from 6 countries. Am J Epidemiol.

[22] Knaus WA, Zimmerman JE, Wagner DP, Draper EA, Lawrence DE (1981). APACHE-acute physiology and chronic health evaluation: a physiologically based classification system. Crit Care Med.

[23] Knaus WA, Draper EA, Wagner DP, Zimmerman JE (1985). APACHE II: a severity of disease classification system. Crit Care Med.

[24] Vincent JL, Moreno R (2010). Clinical review: scoring systems in the critically ill. Crit Care.

[25] Schein M, Gecelter G, Freinkel Z, Gerding H (1990). APACHE II in emergency operations for perforated ulcers. Am J Surg.

[26] Bosscha K, Reijnders K, Hulstaert PF, Algra A, van der Werken C (1997). Prognostic scoring systems to predict outcome in peritonitis and intra-abdominal sepsis. Br J Surg.

[27] Kulkarni SV, Naik AS, Subramanian N (2007). APACHE-II scoring system in perforative peritonitis. Am J Surg.

[28] Ding LA, Sun LQ, Chen SX, Qu LL, Xie DF (2007). Modified physiological and operative score for the enumeration of mortality and morbidity risk assessment model in general surgery. World J Gastroenterol.

[29] Koç M, Yoldaş O, Kiliç YA, Göçmen E, Ertan T, Dizen H (2007). Comparison and validation of scoring systems in a cohort of patients treated for perforated peptic ulcer. Langenbecks Arch Surg.

[30] Malik AA, Wani KA, Dar LA, Wani MA, Wani RA, Parray FQ (2010). Mannheim Peritonitis Index and APACHE II--prediction of outcome in patients with peritonitis. Ulus Travma Acil Cerrahi Derg.

[31] Berreta J, Kociak D, Balducci A, De Feo F, Laplacette MV, Bellido F (2010). Generalized secondary peritonitis: predictors of in-hospital mortality and survival and mortality evolutive links. Acta Gastroenterol Latinoam.

[32] Aslar AK, Ozdemir S, Mahmoudi H, Kuzu MA (2011). Analysis of 230 cases of emergent surgery for obstructing colon cancer--lessons learned. J Gastrointest Surg.

[33] Delibegovic S, Markovic D, Hodzic S (2011). APACHE II scoring system is superior in the prediction of the outcome in critically ill patients with perforative peritonitis. Med Arh.

[34] van Ruler O, Kiewiet JJ, Boer KR, Lamme B, Gouma DJ, Boermeester MA (2011). Failure of available scoring systems to predict ongoing infection in patients with abdominal sepsis after their initial emergency laparotomy. BMC Surg.

[35] Viehl CT, Kraus R, Zürcher M, Ernst T, Oertli D, Kettelhack C. The Acute Physiology and Chronic Health Evaluation II score is helpful in predicting the need of relaparotomies in patients with secondary peritonitis of colorectal origin. Swiss Med Wkly 2012; 142: w13640. 10.4414/smw.2012.13640 22832947

[36] Thorsen K, Søreide JA, Søreide K (2013). Scoring systems for outcome prediction in patients with perforated peptic ulcer. Scand J Trauma Resusc Emerg Med.

[37] Das K, Ozdogan M, Karateke F, Uzun AS, Sozen S, Ozdas S (2014). Comparison of APACHE II, P-POSSUM and SAPS II scoring systems in patients underwent planned laparotomies due to secondary peritonitis. Ann Ital Chir.

[38] Ausania F, Guzman Suarez S, Alvarez Garcia H, Senra Del Rio P, CasalNuñez E (2015). Gallbladder perforation: morbidity, mortality and preoperative risk prediction. Surg Endosc.

[39] V A M, C P M, S S, Srinivasarangan M. Efficacy of Mannheim Peritonitis Index (MPI) Score in Patients with Secondary Peritonitis. J Clin Diagn Res 2014 Dec; 8(12): NC01–3. doi: 10.7860/JCDR/2014/8609.5229. Epub 2014 Dec 5. 10.7860/JCDR/2014/8609.5229 PMC4316291 25653985

[40] Qureshi AM, Zafar A, Saeed K, Quddus A (2005). Predictive power of Mannheim Peritonitis Index. J Coll Physicians Surg Pak.

[41] Boey J, Wong J, Ong GB (1982). A prospective study of operative risk factors in perforated duodenal ulcers. Ann Surg.

[42] Boey J, Choi SK, Poon A, Alagaratnam TT (1987). Risk stratification in perforated duodenal ulcers. A prospective validation of predictive factors. Ann Surg.

[43] Altaca G, Sayek I, Onat D, Cakmakçi M, Kamiloğlu S (1992). Risk factors in perforated peptic ulcer disease: comparison of a new score system with the Mannheim Peritonitis Index. Eur J Surg.

[44] Mishra A, Sharma D, Raina VK (2003). A simplified prognostic scoring system for peptic ulcer perforation in developing countries. Indian J Gastroenterol.

[45] Møller MH, Engebjerg MC, Adamsen S, Bendix J, Thomsen RW (2012). Acta Anaesthesiol Scand. The Peptic Ulcer Perforation (PULP) score: a predictor of mortality following peptic ulcer perforation. A cohort study.

[46] Lawrence S, Friedman MD (2010). Surgery in the Patient with Liver Disease. Trans Am Clin Climatol Assoc.

[47] Teh SH, Nagorney DM, Stevens SR, Offord KP, Therneau TM, Plevak DJ (2007). Risk factors for mortality after surgery in patients with cirrhosis. Gastroenterology.

[48] Alves A, Panis Y, Mantion G, Slim K, Kwiatkowski F, Vicaut E (2007). The AFC score: validation of a 4-item predicting score of postoperative mortality after colorectal resection for cancer or diverticulitis: results of a prospective multicenter study in 1049 patients. Ann Surg.

[49] Fazio VW, Tekkis PP, Remzi F, Lavery IC (2004). Assessment of operative risk in colorectal cancer surgery: the cleveland clinic foundation colorectal cancer model. Dis Colon Rectum.

[50] Whiteley MS, Prytherch D, Higgins B, Weaver PC, Prout WG (1996). An evaluation of the POSSUM surgical scoring system. Br J Surg.

[51] Prytherch DR, Whiteley MS, Higgins B, Weaver PC, Prout WG, Powell SJ (1998). POSSUM and Portsmouth POSSUM for predicting mortality. Physiological and operative severity score for the enumeration of mortality and morbidity. Br J Surg.

[52] Mohamed K, Copeland GP, Boot DA, Casserley HC, Shackleford IM, Sherry PG (2002). An assessment of the POSSUM system in orthopaedic surgery. Journal of Bone & Joint Surgery - British Volume.

[53] Kurita M, Ichioka S, Tanaka Y, Umekawa K, Oshima Y, Ohura N (2009). Validity of the orthopedic POSSUM scoring system for the assessment of postoperative mortality in patients with pressure ulcers. Wound Repair Regen.

[54] Mosquera D, Chiang N, Gibberd R (2008). Evaluation of surgical performance using V-POSSUM risk-adjusted mortality rates. ANZ J Surg.

[55] Ozkan O, Guner A, Kaya U, Kece C, Reis E, Kesici S (2014). Evaluation of CR-POSSUM, original ACPGBI and new ACPGBI scoring systems for colorectal cancer surgery. Chirurgia (Bucur).

[56] Finlay IG, Edwards TJ, Lambert AW (2004). Damage control laparotomy. Br J Surg.

[57] Mohil RS, Bhatnagar D, Bahadur L, Rajneesh, Dev DK, Magan M (2004). POSSUM and P-POSSUM for risk-adjusted audit of patients undergoing emergency laparotomy. Br J Surg.

[58] Brooks MJ, Sutton R, Sarin S (2005). Comparison of Surgical Risk Score, POSSUM and p-POSSUM in higher-risk surgical patients. Br J Surg.

[59] Campillo-Soto A, Flores-Pastor B, Soria-Aledo V, Candel-Arenas M, Andrés-García B, Martín-Lorenzo JG (2006). The POSSUM scoring system: an instrument for measuring quality in surgical patients. Cir Esp.

[60] Ding LA, Sun LQ, Chen SX, Qu LL, Xie DF (2007). Modified physiological and operative score for the enumeration of mortality and morbidity risk assessment model in general surgery. World J Gastroenterol.

[61] Mohil RS, Singh T, Arya S, Bhatnagar D (2008). Risk adjustment is crucial in comparing outcomes of various surgical modalities in patients with ileal perforation. Patient Saf Surg.

[62] Kumar P, Rodrigues GS (2009). Comparison of POSSUM and P-POSSUM for risk-adjusted audit of patients undergoing emergency laparotomy. Ulus Travma Acil Cerrahi Derg.

[63] de Castro SM, Houwert JT, Lagard SM, Busch OR, van Gulik TM, Gouma DJ (2009). POSSUM predicts survival in patients with unresectable pancreatic cancer. Dig Surg.

[64] Luna A, Rebasa P, Navarro S, Montmany S, Coroleu D, Cabrol J (2009). An evaluation of morbidity and mortality in oncologic gastric surgery with the application of POSSUM, P-POSSUM, and O-POSSUM. World J Surg.

[65] Garcea G, Ganga R, Neal CP, Ong SL, Dennison AR, Berry DP (2010). Preoperative early warning scores can predict in-hospital mortality and critical care admission following emergency surgery. J Surg Res.

[66] Yadav K, Singh M, Griwan M, Mishra Ts, Kumar N, Kumar H (2011). Evaluation of POSSUM and P-POSSUM as a tool for prediction of surgical outcomes in the Indian population. Australas Med J.

[67] Sreeharsha H, Sp R, Sreekar H, Reddy R (2014). Efficacy of POSSUM score in predicting the outcome in patients undergoing emergency laparotomy. Pol Przegl Chir.

[68] Glance LG, Lustik SJ, Hannan EL, Osler TM, Mukamel DB, Qian F (2012). The Surgical Mortality Probability Model: derivation and validation of a simple risk prediction rule for noncardiac surgery. Ann Surg.

[69] Shapter SL, Paul MJ, White SM (2012). Incidence and estimated annual cost of emergency laparotomy in England: is there a major funding shortfall?. Anaesthesia.

[70] Nightingale JJ, Burmeister L, Hopkins D (2011). A national survey of the use of epidural analgesia in patients with sepsis undergoing laparotomy. Anaesthesia.

